# Alexithymia and gambling: Psychotherapy to differentiate feelings

**DOI:** 10.1192/j.eurpsy.2021.1322

**Published:** 2021-08-13

**Authors:** A. Savnikova, O. Khaustova

**Affiliations:** Medical Psychology, Psychosomatic Medicine & Psychotherapy, Bogomolets National Medical University, Kiyv, Ukraine

**Keywords:** Gambling, alexithymia, psychotherapy

## Abstract

**Introduction:**

The relationship of alexithymia with gambling addiction is not obvious, but it is present, as evidenced by the results of many studies. Alexithymia is likely to associate with gambling as a coping behavior to increase emotional arousal and avoid negative emotions, according to the affect dysregulation model. Alexithymic individuals experience the same spectrum of emotions as ordinary people, however, from the standpoint of psychology, psychiatry, unexpressed emotions are repressed into the subconscious, and their bodily manifestations accumulate.

**Objectives:**

We plan to conduct research to improve the medical and psychological support of patients with pathological gambling due to the presence of alexithymia.

**Methods:**

A systematic search of the literature was run in the major reference databases including PubMed, Cochrane Database for Systematic Review, Web of Science, Scopus until 2019. All studies assessed alexithymia with the Toronto Alexithymia Scale while gambling problems were assessed mostly with the South Oaks Gambling Screen.

**Results:**

We assume that for pathological gamblers, specific psychotherapeutic techniques like body-centered psychotherapy could help them to differentiate feelings from bodily sensations.

**Conclusions:**

The results highlight the importance of taking in the relationship between alexithymia and pathological gambling. Further studies are needed to widen the knowledge of this association.

